# Use of Aspirin and Statin as primary prevention for cardiovascular diseases

**DOI:** 10.12669/pjms.326.10615

**Published:** 2016

**Authors:** Bahaa Aba Alkhail, Rahila Iftikhar, Adnan Al Shaikh

**Affiliations:** 1 Bahaa Aba Alkhail, Department of Family and Community Medicine, King Abdulaziz University Hospital, Jeddah, Saudi Arabia; 2 Rahila Iftikhar, Department of Family and Community Medicine, King Abdulaziz University Hospital, Jeddah, Saudi Arabia; 3 Adnan Al Shaikh, King Saud bin Abdulaziz University for Health Science, Pediatric Department, King Abdulaziz Medical City-W, MNGHA, Jeddah, Saudi Arabia

**Keywords:** Aspirin, Cardiovascular disease, Diabetes mellitus, Primary prevention, Statin

## Abstract

**Objective:**

To ascertain whether recommendations for the use of Statins and Aspirin as primary prevention in diabetic patients are correctly implemented at our institution.

**Methods:**

This cross-sectional study was conducted between February 2014 and April 2014 at the General Practice Department of King Abdulaziz University Hospital. Three hundred twelve patients were included in the study. Data were collected from the electronic patient medical records for the characteristics of the patients, existing co-morbidities, and results of laboratory investigations. Descriptive statistics were performed for all variables.

**Results:**

Of 312 patients, aspirin was indicated for 17.0% but it was not prescribed. It was both indicated and prescribed in 36.2% of the cases. Statin treatment was indicated in 27.2% of the patients but it was not prescribed, while in 63.1% of cases it was indicated and prescribed

**Conclusion:**

The proportion of patients who achieved treatment targets at our institution is greater than that reported by other studies, albeit treatment targets are not being met in a significant number of cases.

## INTRODUCTION

The burden of cardiovascular disease (CVD) is substantial in patients with diabetes mellitus, who have a two-to four-fold increase in the incidence of cardiovascular events compared with age- and sex-matched, non-diabetic persons.^1^,^2^ The increased risk of cardiovascular events and mortality in diabetic patients has created a need for researchers to identify more efficient strategies for cardiovascular risk reduction.

During the past three decades, several trials have attempted to demonstrate the efficacy of aspirin and statins to decrease cardiovascular events in diabetic patients, albeit with conflicting evidence about the efficacy of aspirin for primary prevention of CVD in persons with diabetes. While the American Diabetes Association (ADA), American Heart Association (AHA), and the American College of Cardiology Foundation (ACCF) created updated recommendations, these guidelines are, in practice, difficult to implement.^3^

In practice, the implementation of guidelines is influenced by various factors and, depending on the circumstances, the target goal is often not easy to achieve. These factors may include physician knowledge and attitudes, patient behaviors, or health system facilities. The ADA guidelines recommend a comprehensive care plan for diabetic patients. These guidelines comprise preventative medication use of aspirin and statins based on specific criteria.

According to previous research,^4^ aspirin and lipid-lowering agents are often not appropriately prescribed: in some practices aspirin is overprescribed, whereas statins are under prescribed. While it is crucial to understand current practices in diabetes care in order to improve outcomes in diabetic patients, there are few data that show to what extent these guidelines are followed.

We aimed to investigate whether recommendations for the use of statins and aspirin in diabetic patients are correctly employed at our institution. Therefore, this study was designed to assess the prescription of aspirin and statin therapy in diabetic patients to prevent CVD.

## METHODS

This cross-sectional, study was conducted between February 2014 and April 2014 at the General Practice Department of King Abdulaziz University Hospital (KAUH) Jeddah, Saudi Arabia. All patients with ischemic heart disease, kidney disease and stroke were excluded.

Data were collected from the patients’ electronic medical records for demographic and clinical characteristics, including existing co-morbidities, and laboratory findings.

We followed the ADA 2014 recommendations,^5^ which recommends initiating statin therapy in individuals with diabetes and other cardiovascular risk factors target LDL cholesterol levels of <100 mg/dL are recommended in this group of patients. The ADA recommends aspirin for those who are ≥ 50 years old or who have additional risk factors (family history of CVD, high blood pressure [BP], smoking, dyslipidemia, or albuminuria). The ACCF recommends aspirin for high-risk diabetic patients at increased cardiovascular risk.

Similarly, we followed the ADA working definition for aspirin therapy:

a) Cardiovascular disease risk >10%,
b) Men aged 50 years or women aged 60 years who have at least one additional major risk factor (family history of CVD, hypertension, smoking, dyslipidemia, microalbuminuria), or
c) Diabetes mellitus with CVD.

### Ethics

Permission to conduct this study was obtained from the Ethics Research Committee at King Abdulaziz University, Jeddah. All procedures followed were in accordance with the ethical standards of the Ethics Research Committee at King Abdulaziz University, Jeddah and with the latest (2008) version of Helsinki Declaration of 1975.

### Statistical analysis

The data were entered and analyzed using the Statistical Package for the Social Sciences (SPSS), version 16. Normal distribution of the data was checked by Shapiro-Wilk’s statistics and if the p-value was ≤ 0.05, the data were considered to be non-normally distributed. Normally distributed quantitative variables are presented as mean ± standard deviation (SD). Frequencies and percentages were calculated for qualitative variables.

## RESULTS

The study included 312 patients who were aged 52 to 67 years (mean, 59.6±11.8 years). Patients aged ≥ 50 years comprised approximately four-fifths of the sample. Females accounted for approximately 72.6% of the sample. The majority of the patients (93.7%) were married. The sample was composed of patients of different ethnic and racial origins, only one-third of which were Saudis. Approximately 77.1% had completed at least high school (Table-I).

**Table-I:** Demographic characteristics of the patients (n=312).

Variable	Frequency (Percent)*
*Age Group*	
≤ 39 years	13 (4.2)
40-49 years	43 (13.8)
50-59 years	107 (34.3)
60-69 years	86 (27.6)
70-79 years	49 (15.7)
80+ years	14 (4.5)
*Gender*	
Male	86 (27.6)
Female	226 (72.4)
*Marital Status*	
Single	10 (3.3)
Married	283 (93.7)
Divorced / widowed	9 (3.0)
*Nationality*	
Saudi	98 (31.6)
Non-Saudi	214 (68.4)
*Educational Status*	
Primary	45 (19.5)
Intermediate	67 (29)
High school	66 (28.6)
Graduate	48 (20.8)
Post graduate and above	5 (2.2)

* For some observations, the total is < 312 due to missing values.

Aspirin was indicated in 17.0% of the 312 patients but was not prescribed; it was indicated and prescribed in 36.2% of the cases. In more than half of the patients (63.1%) statin therapy was indicated and prescribed. However, statin treatment was indicated in 27.2%of the patients but it was not prescribed (Table-II).

**Table-II:** Statin and Aspirin prescription among indicated and non-indicated cases.

Variable	Indicated n (%)	Non-indicated n (%)	Frequency
*Aspirin therapy*			
Prescribed	113 (36.2)	42 (13.5)	155
Not prescribed	53 (17.0)	79 (25.3)	132
Total	166 (100.0)	121 (100.0)	287*
*Statin Therapy*			
Prescribed	197 (63.1)	5 (1.6)	282
Not prescribed	85 (27.2)	8 (2.6)	13

Total	202 (100.0)	93 (100.0)	295*

* The total is < 312 due to missing values.

## DISCUSSION

Based on the recommendations of the ADA, AHA, and ACCF, most of the patients in our cohort were at high-risk for cardiovascular events: approximately four-fifths were aged ≥ 50 years and about 65% had at least two co-morbid conditions. However, 17.0% and 27.2% of patients at increased risk for cardiovascular events did not receive aspirin and statin treatments, respectively. This demonstrated gaps in cardiovascular risk management. Other researchers have reported similar gaps in the management of cardiovascular risk. While aspirin is frequently used by millions of patients worldwide, especially for primary prevention of CVD, many patients with established CVD are not using aspirin.^6^ In another report,^7^ it was found that statin treatment was not prescribed for 41% of diabetic patients at high risk for cardiovascular events.

In this study we specifically considered whether aspirin was indicated for primary prevention of CVD. Recent research demonstrates that its indication in the primary prevention of CVD is very limited. Nevertheless, many physicians tend to prescribe aspirin for primary prevention of CVD without quantifying CVD risk, and it might take some time for physicians to modify their practice in response to current recommended guidelines.^8^ Recommendations for prescribing aspirin to diabetic patients need to be more emphasized, informed and explained to physicians. Physicians should be educated about comprehensive diabetes management and risk reduction strategies in these patients. Gaps between guideline recommendations and practice in managing BP and lipids are relatively high compared with gaps for controlling glucose.^9^ Comprehensive treatment must include blood pressure lowering, tobacco cessation, reaching optimal weight and emphasizing on regular walks for primary atherosclerotic cardiovascular disease (ASCVD) prevention.

It has been shown that Diabetic care is not optimum. A recent report from Italy indicates that only 46.3% of high risk CVD patient used aspirin for prevention,^10^ while in another study 35% of clinicians indicated that patients were using it for primary prevention.^11^ Similarly, a survey showed a small proportion of patients were prescribed aspirin.^12^ Conversely, another study demonstrated overutilization of aspirin in patients for whom the drug was not recommended.^13^ In fact, aspirin should be prescribed appropriately,^14^ and given that the risk of gastrointestinal bleeding must be weighed against the potential benefit, physicians should make shared decisions.^15^ Aspirin utilization and statin prescription can be improved by using a heart scoring system.^16^ Furthermore; this scoring system can enhance appropriate targeting of high risk population.

We found that 63 %of patients were prescribed statins appropriately. In another study that assessed statin prescribing patterns according to the ADA guidelines, only 35.1% of the patients were prescribed statins.^17^ The new ACC/AHA guidelines recommend primary prevention with moderate-intensity statin to individuals with diabetes aged 40-75 years and LDL-C 70-189 mg/d. This will definitely increase the number of patient eligible for statin treatment. Although statins carry a small risk for new onset DM, clinical guidelines recommend its use in high risk groups, as the benefits outweigh the risks.^18^

### Limitations of the study

First, this single-center study was conducted in a tertiary care setting. Hence, the results cannot be generalized to the local population. Second, the patients in our study had free health care, including dietician consultation, and medication, which might not be available to patients in other settings. Third, we did not perform inferential statistics to determine whether differences between patients were significant. Finally, we did not discuss the complications of diabetes. We, therefore, suggest that future studies should investigate and discuss the effectiveness of aspirin and statins in the primary prevention of CVDs in patients with diabetes.

## CONCLUSION

These findings reveal that our patients who had good glycemic control, the use of aspirin and statin for the prevention of CVD in diabetic patients can be improved. This suggests the need to investigate the reasons for this failure in order to develop strategies for awareness program and a better multidisciplinary approach to achieve treatment targets. We also suggest further investigations to better understand the basis for physicians’ estimates of how many of their patients with diabetes were reaching target values, as this will help to evaluate current practice trends and assist in formulating programs to enhance the appropriate use of primary prevention strategies in our setting.
